# Empathic and Empathetic Systematic Review to Standardize the Development of Reliable and Sustainable Empathic Systems

**DOI:** 10.3390/s22083046

**Published:** 2022-04-15

**Authors:** Karl Daher, Dahlia Saad, Elena Mugellini, Denis Lalanne, Omar Abou Khaled

**Affiliations:** 1 HumanTech Institute, HEIA-FR, University of Applied Sciences Western Switzerland HES-SO, 2800 Delémont, Switzerland; dahlia.saad@edu.hefr.ch (D.S.); elena.mugellini@hes-so.ch (E.M.); omar.aboukhaled@hes-so.ch (O.A.K.); 2 Human-IST, University of Fribourg, 1700 Fribourg, Switzerland; denis.lalanne@unifr.ch

**Keywords:** empathic, empathy, emotion, detection, response, modalities

## Abstract

Empathy plays a crucial role in human life, and the evolution of technology is affecting the way humans interact with machines. The area of affective computing is attracting considerable interest within the human–computer interaction community. However, the area of empathic interactions has not been explored in depth. This systematic review explores the latest advances in empathic interactions and behaviour. We provide key insights into the exploration, design, implementation, and evaluation of empathic interactions. Data were collected from the CHI conference between 2011 and 2021 to provide an overview of all studies covering empathic and empathetic interactions. Two authors screened and extracted data from a total of 59 articles relevant to this review. The features extracted cover interaction modalities, context understanding, usage fields, goals, and evaluation. The results reported here can be used as a foundation for the future research and development of empathic systems and interfaces and as a starting point for the gaps found.

## 1. Introduction

*Empathy is about finding echoes of another person in yourself.—Mohsin Hamid*

This was Mohsin Hamid’s (https://www.newyorker.com/books/page-turner/this-week-in-fiction-mohsin-hamid, accessed on 23 February 2022) definition of empathy when he attempted to find a boy’s echoes in a story back in 2012. It falls within the scope of the main scientific definition, proposed in 1897 by Theodor Lipps, who defined empathy as the “feeling into” [1], where Mohsin Hamid attempted to understand within the boy’s feelings. Today, empathy has more than forty-three different definitions in the research [2]. The concept of empathy is difficult to understand, particularly with the existing diversity of the definitions. Thus far, there is no unique determined justification for empathy that clarifies its meaning, usage, and importance.

However, empathy is an important factor between humans; it is when a person attempts to see a problem from the other’s perspective. As described by Seïler and Craig, “it is an interaction between two individuals who share each other’s experiences and feelings” [3]. Therefore, empathy is a tool to enhance relationships between humans and plays a crucial role in the collaboration between individuals [4]. Currently, with humans spending more time on machines, the need for empathy in machines is crucial. A machine should be able to understand and react correspondingly to certain actions coming from the human, taking into consideration their emotions and the context.

However, just as empathy is between at least two humans, empathy with machines cannot only be one-sided. It is an interaction cycle between the human and the machine, creating a complete loop of detection and response between the two. Detection and response are included in human genes, where the former is the comprehension of others’ bodies [5] and facial responses [6] in addition to capturing their vocal expressions [7], while the latter reacts through our body language and facial expressions and is expressed through our voice capabilities.

In the case where the interaction is between a human and a machine, the detection and response interaction modality needs to be adapted depending on the context just as with humans. For a more comprehensive interaction, the machine should use different detection algorithms and different response modalities, where, as with humans, these response modalities need to be adapted to the context of the situation as well. For example, a machine making loud noises at midnight would not be the most suitable response at that moment when a small text would be enough, unless, for example, it is an emergency. Thus, having multiple modalities adapted to different situations is a significant challenge.

Yet, when speaking about interaction modalities and user interaction, user interfaces are intermediate between the human and the core of the machine. User interfaces have evolved over the years, moving from levers and buttons to Graphical User Interfaces (GUI), and ending up as Tangible User Interfaces (TUI). However, important features are still missing in these user interfaces, as neither empathy nor emotions were considered when user interfaces were first created. Today, empathy and emotions need to be taken into consideration and be integrated in a new type of interface. Lately, fast development has helped in including emotion and empathy, yet there is no actual standardisation of empathic systems because of the diversification of empathy.

We present a systematic review of systems that have empathic or empathetic behaviour. The review analysed articles from the ACM Conference on Human Factors in Computing Systems—CHI conference between 2011 and 2021, as being the most prestigious human–computer interaction conference. We extracted features related to the creation of interfaces that have an important effect on empathy and emotions within machines in the future. The features extracted put together empathic system behaviour from the detection and response standpoints.

We further discuss the features extracted from the articles and the conclusions drawn from the findings of this systematic review. We finish with real-life examples where systems, applications, and interfaces can be created or modified to fit and become empathic. The motivation behind this work lies behind the research of the authors on empathy in machines and on the effect of machine empathic responses. The incomplete state of the art in the consideration of emotions for developing new technologies and interfaces is a crucial motivator behind this work. Combining a concept and the modelling of empathic interfaces will help future research move toward standardisation and the creation of fundamental models.

## 2. Related Work

Empathy plays an important role in human life. It is the intersection between humans. Empathy helps humans bond, understand each other, sympathise with each other, and instigate collaboration [4,8,9]. With the fast spread of machines, empathy integration is becoming beneficial. Currently, humans who tend to acquire trust from a machine would be employing the concept of empathy in their behaviour [10,11]. Empathy is one of the main topics in the affective computing domain. Affective computing was presented by Rosalind Picard as a computer algorithm and a model of recognition based on human emotions [12].

Researchers found that there is a lack of resources and databases for body language and voice, for example, leading to low accuracy and the inability to generalise the methods used [13]. Since then, the affective computing topic has propagated to many sub-fields, such as facial expressions, body language, voice recognition, physiological signals, and many more. Today, affective computing is reaching new standards and overcoming barriers, particularly with the integration of artificial intelligence. Where the development is now evolving toward integrating abstract domains, such as empathy in agents instead of emotions. Recently, an affective avatar was developed by adding emotional intelligence and emotion expression through facial expressions and was considered a reliable associate to work with by users [14].

From here, we cover the importance of understanding the state of research on empathy integrated into agents and the cycle of detection and response applied in an ethical, standardised, and suitable way.

Empathy as detection and response. Empathy between humans is based on communication between two people, the emotions they share, and the links they create. There exist three types of empathy defined in the state of the art considered as the main classification: cognitive empathy, affective empathy, and compassionate empathy. Cognitive is the recognition of the transmitter’s feelings by the receiver, affective is when the receiver feels the transmitter’s feelings, and compassionate is when the receiver has the urge to help the transmitter [15].

Other researchers defined other types of empathy and clustered them into perfect empathy, truncated empathy, and contaminated empathy [16]. The dissection of empathy and determination of its main components was studied by Janssen, where three main components were identified, which are broadly agreed upon as stated in the psychology and neuroscience fields.

Janssen’s three components are cognitive empathy, where the receiver deduces what the transmitter is feeling; emotional convergence, where the receiver experiences the transmitter’s emotions; and empathic responding, where the receiver’s response is sympathy or personal trouble [17]. Thus, empathy components and divisions are not yet standardised nor clearly partitioned, even though empathy is an important factor in daily life. Empathy can have a great effect on humans’ mental and physical health; therefore, before creating a new application, a few important points need to be taken into consideration.

Anyasodo and Burnett, after their experiments with professionals in creating mental models of empathy, defined important themes that can have a higher impact if taken into consideration. For example, empathy occurs when the receiver recognises the emotional state of the transmitter and what they are attempting to express [18]. This can be seen in the Delight experiment where the information is transmitted using ambient light, which makes the human receive and understand the request transmitted by the machine [19]. Another Anyasodo mental model was represented in the Delight experiment, where the interaction between transmitter and receiver is based on the output and not on the detection/response cycle for it to be considered empathic [18].

Sharing emotions and having empathic behaviour can improve well-being, as can already be seen in experiments conducted between humans for using empathy in the mindfulness-based stress reduction MBSR program [20]. Thus, creating new technologies that possess these characteristics can increase the quality of life and monitor different human aspects.

From this, we can see the importance of empathy and the necessity of having two parties for it to be initiated. Empathic interaction works as a detection and response cycle between two sides. Detection and response play an important role in the communication between the two parties. The communication can be either verbal or nonverbal. A verbal response can be through voice, text, and text-to-speech, while a nonverbal response can be through light, music, and visuals.

To apply detection and response in human–computer interactions, the interfaces are the layer between the human and the core of the machine.

User interfaces as an interaction layer between humans and machines. The interactions between humans and machines occur in a space called the *User Interface* or UI. The goal of UIs is to improve the efficiency and accuracy of these interactions by allowing humans to easily and effectively manipulate and control machines, while the latter simultaneously sends back information that facilitates decision-making for the operators. Graphical User Interfaces—GUIs are the most common and dominant UI, while Tangible User Interfaces—TUIs are emerging interfaces developed to overcome GUI’s weaknesses. GUIs emerged with the role of facilitating human–computer interactions by removing obstacles in user-computer system communication and allowing users to focus on the task at hand [21].

They represent data and information in a visual format on monitors, and they manipulate these using remote controllers, such as a mouse, a keyboard, or any other type of input peripherals. However, even though GUIs are flexible enough to represent a variety of media, they still lack realism, the use of the sense of touch, and involvement in everyday life [22]. TUI was introduced as the “Graspable User Interface” in 1995 [23]; however, due to the publication of studies on human–computer interaction, the new term “Tangible User Interface” was introduced [24].

TUIs allow users to interact with computers using graspable physical objects. These objects are a representation and are used to control digital data. TUIs support multi-user interactions since many users can interact with the tangible system simultaneously, which is considered an advantage compared to GUIs where peripherals can allow only one user to interact with the computer at a time [25]. Identifying the user’s emotion in UI helps provide a useful evaluation of the means used to achieve user goals in the interface.

Being familiar with emotions can also help to understand user–machine interactions and the user response during these interactions to, thus, perform a dynamic and intelligent adaptation [26]. Both GUI and TUI still lack acknowledgment of user emotions while interacting with the machine: the absence of emotion detection leads to the absence of an empathic response. Therefore, and since humans are driven by emotions and empathy, new Empathic User Interface (EUI) designs are now in development. One of the important roles of a EUI is in detecting and precisely collecting emotions during interactions.

There exist two methods for collecting emotions: implicit and explicit. The former, also named the objective method, collects the user’s emotional responses without asking the user, by measuring and reading their behaviour, expressions, and physiological and neuropsychological activities. The latter, also called the subjective method, analyses and interprets the emotions given by the user himself and by an observer [26].

We analysed the latest works that have been conducted on empathy, empathic behaviour, and interaction more precisely. In the next section, we show the protocol used for article identification. Furthermore, we show the different parts of empathic behaviour through different extracted features. We present each of the features extracted from each of the articles chosen for this study. We show and discuss the findings of this systematic review. We conclude the article with some examples from real life and propose adaptations to take into consideration empathic behaviour.

## 3. Protocol

In this section, we present, in detail, the strategy of identification and selection of relevant articles as well as how the necessary data were extracted from each chosen article. We also define the features used for the data extraction and add the calculated reviewers’ agreement rate.

### 3.1. Paper Identification

The Association for Computing Machinery (ACM) Conference on Human Factors in Computing Systems, or CHI, is a series of academic conferences in the field of human–computer interaction (HCI) and is considered to be the largest and most prestigious conference in computer science for HCI. Therefore, the database used for this review was the ACM digital library. The keywords used for the search included the terms “empathic” and “empathetic”.

**Figure 1:**
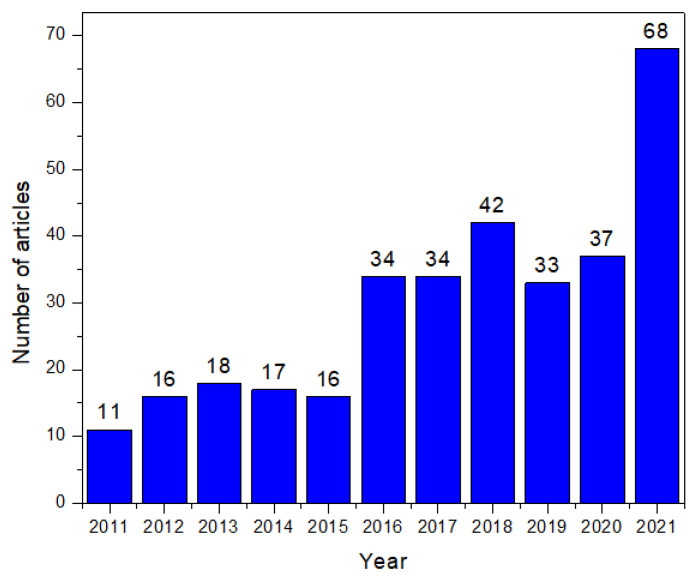
Number of empathic and empathetic articles per year (duplicates excluded).

Both words have the same meaning and are derived from the word empathy. “Empathic” is the original word and was first used in 1909, while “empathetic” is its variant and was first used in 1932. Over the years, the word “empathic” became the word commonly used in scientific writing. The articles chosen were limited to those released between 2011 and 2021, published in the English language and with full-text availability. Based on the above-mentioned terms, a total of 361 articles were found (Figure 1), from which 35 were duplicates.

### 3.2. Paper Selection

**Figure 2:**
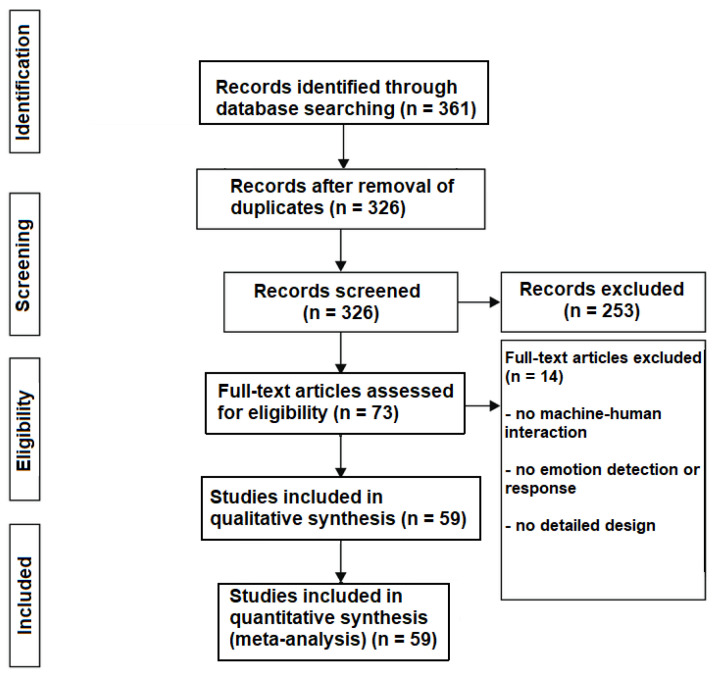
PRISMA flow diagram.

The remaining 326 articles were filtered based on title and abstracts, and 253 articles were excluded, thus, leaving 73 full-text articles to be assessed for eligibility. As a first step, each article reporting an empathic system or analysing emotions was considered eligible. (Figure 2).

The article titles were input into a Google Documents spreadsheet along with the year of publication, and the person assigned to the screening of each article. Two reviewers had access to the Google docs and worked on the evaluation of the obtained articles. Each of them covered half of the selected articles and read them thoroughly to exclude the irrelevant ones.

Papers were considered eligible if they reported a human–machine interaction, an emotion-detection system, the use of a verbal or nonverbal emotional response, a design of the empathic system to be developed or already implemented, and a quantitative or qualitative evaluation of empathy or the empathic system.

Papers were excluded if they did not report a design of an empathic system or machine. The majority of the excluded articles presented human–human interactions, such as interactions between designers and users [27,28,29,30,31,32,33,34,35,36,37], and between medical crowdfunding beneficiaries and contributors [38], and others did not report an empathic system.

### 3.3. Data Extraction

**Figure 3:**
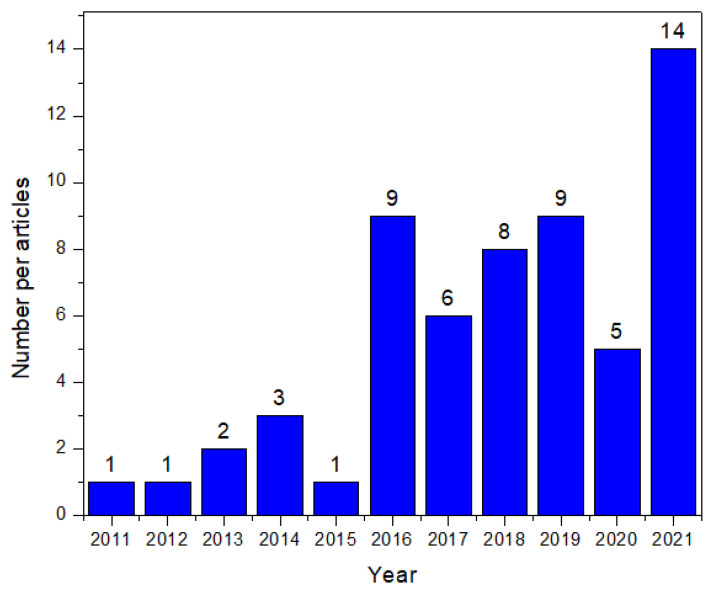
Number of final articles chosen and analysed per year.

For extracting the necessary information from the articles, a table was created in a new spreadsheet. It encompassed the following features: article title and year of publishing, emotion detection and response modalities of the system, subject context semantic analysis (which incorporates the detection and analysis of context, change of context/adaptability, and modification/use of new behaviour techniques), the goal of the system, the automation level changes, the research field of each article, qualitative and quantitative assessment of empathy in addition to any other assessments used, the definition of empathy and the findings from each article, a “design and/or implementation” section, and finally a comment section for reviewers to add their own views regarding each article. After filling the table with the appropriate information, 14 articles were excluded as they did not provide the necessary data required, and 59 were left for the final synthesis (Figure 3 and Table 1).

Before starting with the final assessment, five articles were chosen to test the reviewers’ agreement rate. For each article, features similarly assessed by the reviewers were assigned the number “1”, while features assessed differently were assigned the number “0”. After that, the sum of the assigned numbers was calculated and then divided by the number of features for each article to calculate the agreement rate. Overall, the average reviewer’s agreement rate was 82%.

### 3.4. Features Definition

Definition of Empathy: Empathy does not have a single and standardised definition as we mentioned in Section 2, and each article defined empathyin its own way. Therefore, we gathered the distinct definitions presented in the articles to better understand the authors’ perception of empathy and the influence on their system.

Emotion Detection: Emotion detection is an important part of an empathic machine, as it allows the system to understand and interpret the emotional state of the human. The system can detect a human’s emotions during interactions through verbal or nonverbal communication. Verbal communication consists of voice, text, or voice-to-text. The system uses the tonality, amplitude, and tempo of the voice combined with the words used for analysis. On the other hand, nonverbal communication includes head and body gestures, facial expressions, and physiological signals. Emotions are detected through the changes that occur in these gestures, expressions, and signals.

Emotion Response: Emotion response is another main component of an empathic machine since it shows the abilities of the machine in responding to humans. An emotion response can be made verbally or nonverbally through multiple modalities. The verbal consists of voice, text, and text-to-speech, while nonverbal consists of the usage of light, music, and visuals. Visuals are represented by images, movements, 2D-3D graphs or holograms, etc.

Context Analysis: The context analysis consists of an empathic system’s ability to understand the subject initiated by the user. The system needs to generate the appropriate empathic response. The analysis implies words shared through text or voice, voice tonality and frequency, facial expressions, body gestures, etc. We define three important factors:

- Detection of Context: An empathic system should be able to recognise human emotions regarding a certain subject. To respond in the best possible way, the system needs to analyse and understand the context.
- Change of Context/Adaptability For better self-expression, an empathic machine should be able to understand human perception and expectations. Changing the context or adapting it based on the human’s desires can lead to better understanding and smoother interactions. For example, an empathic system should be able to tell that responding by a text will not result in the desired outcome when dealing with a human in rage; therefore, it should adapt the response modality.
- Modify/Use New Behaviour TechniquesThe empathic system should be environmentally and socially aware of the situation of the user. Thus, using new behaviour techniques is a must. Interruptions, notifications, and warnings are important factors that can be used in an empathic system.

Goal: The goal represents the purpose for which the system was developed. We classified the goals as awareness or solutions. For awareness, the system analyses the human’s reactions within a situation, and the response consists of sensitisation—for example, in dangerous situations. For the latter, the system proposes solutions to overcome problems, such as mental breakdowns or stress management.

Automation Level Changes: The automation level changes consist of the ability of the machine or system to increase or decrease its automation level. An empathic system should—based on the situation—be able to convert and switch between multiple levels of automation. At the same time, the user should also have the ability to manually change the automation level—for example, in the case of autonomous cars. However, not all systems will present the same proprieties; therefore, we attempted to extract any similar behaviours that we detected.

Design or Implementation: The role of this feature is to check whether the study was a design process or an implementation. The design represents the plan with the specifications of the system and studies of the user interface design, while the implementation refers to building the functionalities and testing the user experience.

Field: The field feature represents the domain in which the empathic machine was used. An empathic system could be implemented in any type of field, and this will give us an overview over the fields that have already been studied and what is still missing.

Assessment: The assessment feature was used to show the evaluation of empathy. The assessment was divided in two:

- Quantitative Any system that provides empathy or emotion needs to be quantitatively represented to understand its impact. Thus, for the quantitative assessment, we researched the formulas and mathematical representation of the empathy level. We attempted to find unified models or algorithms for quantitative calculation.
- Qualitative The empathic system directly impacts humans; therefore, qualitative assessment is important to obtain feedback from users. Therefore, we investigated surveys or questionnaires that could reflect the system’s impact. We attempted to find standards that were used or already existing questionnaires that reflected the empathic quality of the system.

Other assessment: We attempted to extract all types of other assessment that the authors took into consideration, so that, in the future, we can add new features that have affected the empathic system or missing scales from existing questionnaires.

## 4. Findings and Results

The analysis of the articles gave us an overview of the different features defined in Section 3.4. First, we present the different definitions of empathy presented by the articles analysed. Then, we present the different findings for each of the features extracted from the articles. We apply our analysis to the design and implementation of an empathic system. Finally, we present the findings of each of the articles in respect to the empathic impact. We discuss the findings in Section 5.

### 4.1. Definition of Empathy in the Articles

*Empathy* is derived from the German word *Einfühlung* meaning “in-feeling” or “feeling into”. It was first translated into English in 1909 by Edward Bradford Titchener. The exact definition of the word empathy is the subject of many debates; therefore, different interpretations of that word have been used in different research papers. For this review, a total of 59 articleswere chosen, presenting different definitions of empathy.

One article used the definition of Alfred Adler for empathy as “seeing with the eyes of another, listening with the ears of another, and feeling with the heart of another” [39]. In another paper, empathy was defined as the ability to understand, discover, and predict an individual’s feelings and emotional state and to respond to them emotionally and compassionately or to experience them indirectly [40,41,42]. Empathy was also described as a combination of empathic concern and personal distress. Empathic concern is the act of feeling for another person rather than feeling for oneself, and personal distress is the feeling of anxiety and discomfort provoked by the distress of others [43].

Additionally, some researchers consider empathy to have two states, cognitive and affective [44,45,46], while others add a third state, compassionate [47,48,49]. Affective empathy is the recognition of another person’s emotions using facial expression, body gestures, voice frequency–tonality–tempo–amplitude, feelings, synchronizing or mimicking those same emotions as a direct result of the person’s affective state. Cognitive empathy is the mental representation and mirroring of the mental state of another person and the understanding of their emotional experiences without having to actually experience their feelings [46]. Compassionate empathy is the feelings of concern, sympathy, and compassion as well as the expression of these feelings toward others. It is normally associated with positive outcomes, such as charitable behaviour and is also considered a result of two other states of empathy [47].

One article [50] defined empathy as four distinguished psychological states: two cognitive and two affective empathic states. The cognitive states are the Imagine-Self perspective, where a person imagines how they would feel and think in the situation of another person, and the Imagine-Other perspective, where a person imagines how another would feel or think in their own situation. The affective states are Emotion Matching, where a person feels the same feelings as another, and Empathic Concern, where a person feels for another in need.

On the other hand, two of the articles chosen for this review used specific types of empathy: situational empathy [51], which is understanding the feelings, emotions, and actions of others during interactions as well as responding immediately to the situation evoked and empathy for animals [52]. The latter includes cognitive empathy for animals, emotional empathy, which is the affective responses to the observed animals’ state, and perceived similarity between animals and humans as well as recognising connections between their behaviours.

### 4.2. Systematic Review Articles Analysis results

In this section, we present the articles and map them to the detection and response modalities. Furthermore, we differentiate between the verbal and nonverbal detection and response behaviour of each of the systems. More detailed analysis is performed over voice-to-text, voice, text, body language, physiological signals, and facial-expression detection modalities. For the response, we extracted text-to-speech, voice, text, light, music, and visuals. We show the distribution of the articles over the detection and response factors and the different types of modalities used. We show the distribution of the article on a 2D map. Then, we further extend the analysis for the other extracted features mentioned in Section 3.4.

#### 4.2.1. Detection and Response Modalities

Designing and developing an empathic system is based on implementing and synchronising the different modalities that a machine can have. The ideal system will have multi-modal interaction patches that can be used to communicate with the user. In this analysis, we attempted to compare the work of researchers to understand the current state of research. The analyses were based on the interest of the research in the detection and response factors mentioned above.

**Figure 4:**
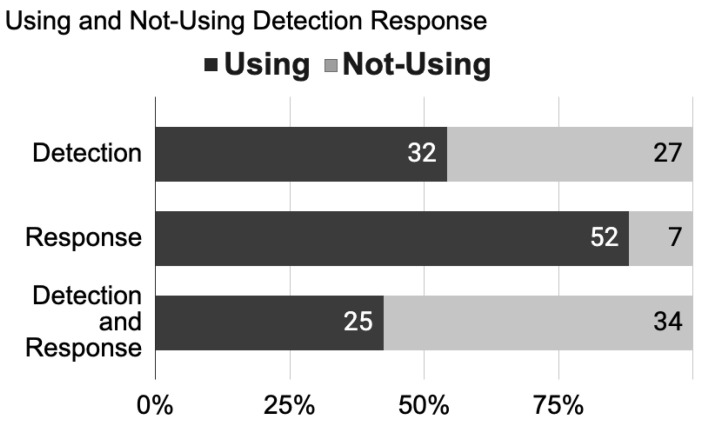
Percentage of articles using Detection, Response and both.

Figure 4 is an overview of the articles analysed in this systematic review, showing the percentage of work conducted on the detection modalities and response modalities. The articles analysed show that 55% of the researchers worked on the detection factor. On the other hand, we can see that 88% of the researchers worked on the response factor. We can conclude that the work was concentrated on the response modality.

In an empathic system, the interaction is based on communication cycles between humans and machines. Thus, a system that works on detection or response alone will not be able to initiate empathic behaviour. In Figure 4, only 42% of the articles used detection and response at the same time. Having a closed loop of interaction between the human and the machine. The remainder of the articles focused on one factor and only on one side of the interaction.

**Figure 5:**
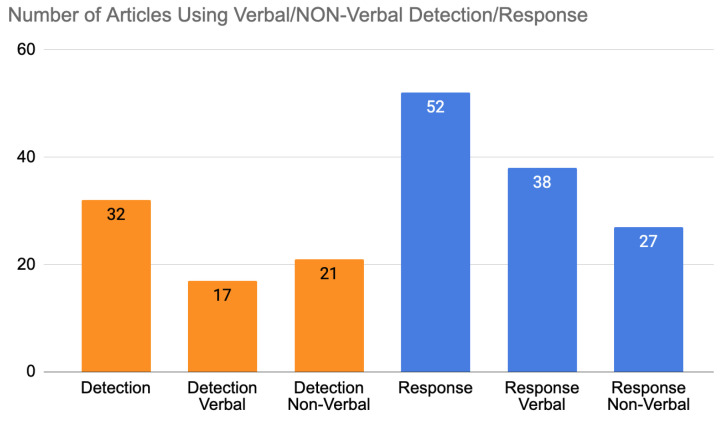
Number of articles using Verbal/Non-Verbal Detection/Response.

A more detailed analysis of detection and response behaviour was made, and this is shown in Figure 5. The results show the number of articles that used verbal and nonverbal detection on one side and the verbal or nonverbal response of machine behaviour on the other. Note that some of the articles used both verbal and nonverbal detection and/or response. The division of the articles shows 32 articles that used detection while 52 used the response out of 59 total articles. Of these 32 detection articles, 17 articles used verbal detection, and 21 used nonverbal detection. From the response articles, 38 used verbal responses, and 27 used nonverbal responses.

**Figure 6:**
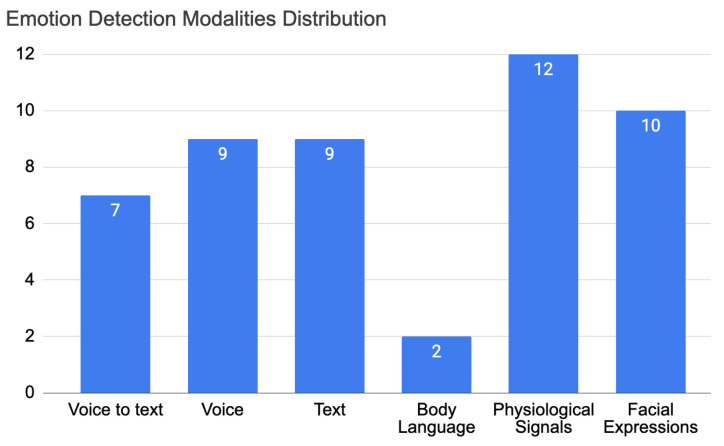
Distribution of articles over the detection modalities.

Looking for more details, we present in Figure 6 the distribution of the articles for the detection factor in each of the modalities. This distribution shows the number of articles that used a certain detection modality. The detection modalities are presented in Section 3 and are defined as voice-to-text, voice, text, body language, physiological signals, and facial expressions. There has been more interest in physiological signals, voice, text, and facial expressions. Body language was the least taken into account for emotion detection factors.

**Figure 7:**
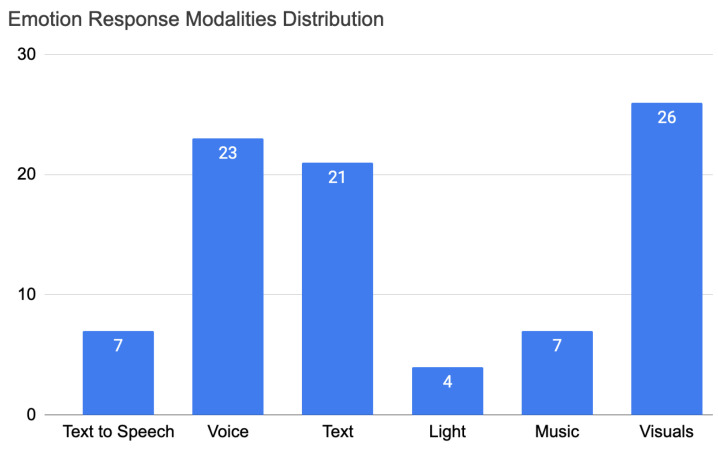
Distribution of articles over the response modalities.

The same analysis was made on the distribution of the articles on the response modalities, and this is shown in Figure 7. This distribution shows the number of articles that are using a certain response modality. The response modalities researched were text-to-speech, voice, text, light, music, and visuals. From this analysis, we notice that visuals had the highest number of articles—26. Visuals vary from upper body movements and gestures [53] and lip syncing with facial expression [54] to a 2D game application [55,56] and abstract and numerical data visualisation [57]. Voice and text were used in 23 and 21 articles, respectively.

**Figure 8:**
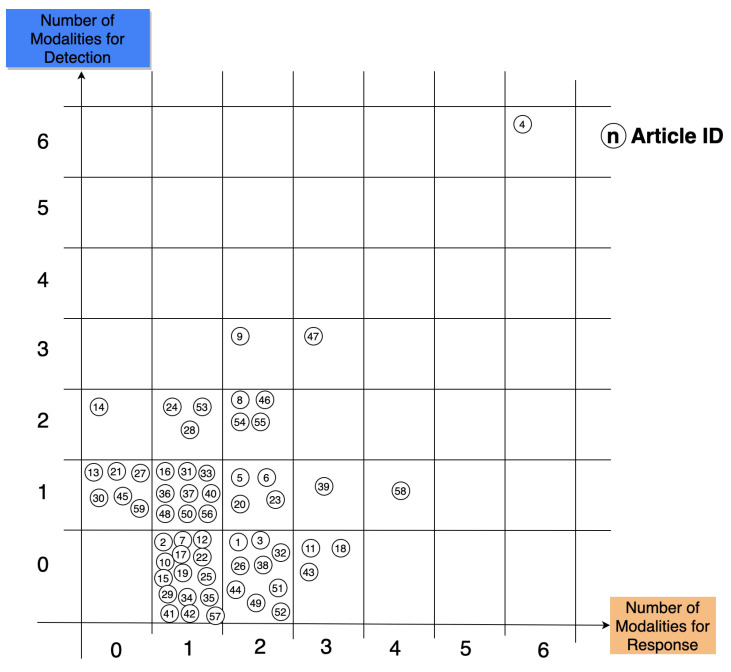
Article representation over detection and response modalities. (number=article_id).

Figure 8 reveals a detailed distribution of the articles analysed in this research over the modalities used in each. The x-axis shows the number of modalities used for the response of the system, while the y-axis shows the number of modalities used for detection. The result shows a high concentration over the little number of modalities between (0,1) and (2,2). Here are some of the class results:

- For zero detection and one response, we have 15 articles working on the response part.
- For one detection and zero response, we have six articles working on the detection part.
- For one detection and one response, we have nine articles working on these types of modalities.
- For zero detection and two responses, we have nine articles working on these types of modalities.

We found a high number of articles working one one response modality, with 15 articles being within this class. One exceptional author mentioned, in their article, six modalities for detection and six modalities of responses. To note, this article was based on the design of an interactive model. There was no implementation of any sort nor any sort of evaluation. This article presents an ideal and complete work of an empathic interface where every modality is included. One article had one type of detection and four types of response, and another used three modalities for response and three for detection. All other articles had a lower number of modalities. From this graph, we can see that the research and development at the current state still falls short compared to human-to-human interaction. Human–machine interactions still have a long way to go before reaching the human-to-human interaction level.

Yet, a high number of modalities does not mean a better empathic system; the result is crucial and is based on the global context, the message interaction modality, the content of the message, and the message context detection.

#### 4.2.2. Further Details

We attempted to further analyse the articles; thus, we extracted more details for each of them. We attempted to analyse the behaviour of the systems proposed in each of the articles. An important factor to understanding the status of the research is to check if the study was a design process or an implementation. Implementation can reach quantitative results and give the effect of each system on humans. Another important feature is the context; contextual understanding was researched in the articles studied to investigate if it was taken into consideration. The goal of the study was extracted in addition to the field in which it was applied. The assessment of empathy is another important feature; we extracted quantitative and qualitative assessments in addition to any other types of assessment.

##### Context Analysis

For the context analysis, the extracted features were the following: the detection of the semantic context, the ability to change the context, and changes in the behaviour modalities.

- Detection of context:
  A total of 21 articles out of 59 detected the context of the situation. This detection is when a machine is able to understand and detect the human’s needs. The context can refer to a behaviour needed within a certain situation used by multiple studies [48,58,59,60,61,62,63,64,65]. Hugo, the companion, was able to detect and save the participant’s queries and requests [58]. Murphy, a robot that accompanies children, can determine the needs of the children according to the context they are in [59]. For schools and students, a design that detects the quietness of a group of students and detects when they go off topic during a group session was developed [60]. Furthermore, imitating human behaviour in groups can also be used when detecting context, particularly when replacing a human by a human-controlled robot. The discussion context between a group of students was captured by the telenoid in a classroom activity [61]. Human behaviour can be used in many situations—for example, for car interfaces where the car can detect good and bad behaviour of the driver and act upon it [62,65]. Eyecams are able to detect sleepiness in human eyes and mimic it to notify the user [63]. Some bots can detect speech to indicate hate speech and improve the ethics of chatbots [64]. Others are designed to detect a transaction risk and inform the user of fraud detection [48]. Alternatively to human behaviour needs, the emotional context in certain scenarios can have a greater impact [39,47,54,66,67,68,69,70]. In other designs, the machine detected the context of emotions to share it with other participants to increase empathy and facilitate discussion [47]. The context of emotions was further improved in psychology where it was used in the modelling of human emotions into vector spaces to detect small changes [66]. Enabling the senses context of another human can improve the empathic connection between the two.
  Empathy Glasses share the senses of another human to understand their perspective by seeing, hearing, and feeling a real world task [39]. On the other hand, Social Signal Processing is used to detect nonverbal cues to share between patients and clinics [67]. Other studies concentrated on the detection of the voice tonality and the emotion being expressed [68]. The multi-modal embodied virtual agent can detect the face pose and emotions shared by landmarks on the user’s face to improve its expressiveness [54]. Bots can be used to detect the psychological state of the user [70]. Empathic GPS systems detect the user state to be able to modify their voice tonality [69]. In a more visual context, pictures are a significant element for context detection [71,72,73,74]. Pictures can be used to share autobiographical memories with others, while lifelog photos presented to the receiver can increase emotional sharing with the transmitter [71]. Further magnetic bookmarks were used to define a certain scenario and tell historical stories, and this type of context detection helps to identify and select the story chosen to be told [72]. While using the scene features is an important factor, visual sentiment and facial features have more influence on the sentiment variation of responses in making conversation with images [73]. Visuals can further be used in face detection—for instance, a driver’s face context can be used to maintain high security of a vehicle by protecting it from intruders and thieves [74].
- Change of Context/Adaptability:
  A total of 6 articles out of 59 changed context in their experiment. The concept is the machine’s ability to adapt to the situation of the human, which can be through adapting the content of the response based on the receiver [47,54,59,68,69,74]. The change of context can be seen in the Voight–Kampff machine, where its role is to change the context of the response sent by adapting it according to the receiver [47], similar to HUE [68], which listens to the user response. SIVA is capable of changing its response based on the facial expressions, head pose, and user voice [54], similar to the adaptability of the voice of Empathic GPS when it feels the user’s physiology [69]. Regarding the context of adaptability, Murphy the miserable robot changes its context based on a determined circumstance of being alone with children or within a group of children [59]. Finally, Doo-Boo changes the context and adapts its behaviour based on the scenario by applying different interaction methods, first, by barking at intruders for security reasons; second, by sharing feedback and assisting the driver by using its tentacles and facial expressions; and third, by sharing indications and using pet-dog characteristics to influence the driver [74].
- Modify/Use New Behaviour Techniques:
  A total of 10 articles out of 59 changed their behaviour techniques. This concept of changing behaviour techniques can have a different impact on humans by sharing in different ways and changing based on human perception. The effect of changing behaviour is not always a positive impact. This change of behaviour can be used in different interaction methods, using notifications [47,75,76], interventions [59,62,76], reminders [44,58], movements [54,74], adaptive behaviour [54,68], etc. Notifications were used as an interruption factor for people performing a defined task to understand its effect based on the task workload [75].
  In addition, a notification was used to share certain information with the user as an incoming chat request in the Voight–Kampff study [47], or as a notification that the text shared by the transmitter is considered offensive in the Feelbook study [76]. In addition to notifications and to recall information about uncompleted tasks, Hugo used reminders to share with the user that their work had not yet been performed [58]. Furthermore, reminders were used to assess the feelings of viewers or as a note explaining the evaluation in another scenario [44].
  Furthermore, the Feelbook application used direct and indirect interventions as a method to detect cyberbullying [76], while Murphy used it to change a child’s behaviour by explaining certain situations differently and by adding positive means into the discussion (for example, a doctor visit) [59]. On the other hand, interventions can also be used when detecting changes in emotional behaviour, and relevant types of interventions need to be addressed based on positive or negative user behaviour, which is the case for vehicles with an empathic car interface [62].
  Body movements and gestures were used for emotional expressiveness in their virtual agents when teaching Tai Chi to enhance learning experiences [54]. Similarly, Doo-Boo shared emotional expressions by waving its tentacle when meeting the driver with happiness or shaking it aggressively when intruders were in the car; furthermore, it was used to maintain the driver’s focus or to point out important information [74].
  For adaptive behaviour, HUE distracted the user when they were frustrated, and used interventions to express empathy when the user shared negative emotions about the next task [68], while SIVA adapted its style of conversation and expression based on the emotional state of the user [54].

##### Goal

For the goals of the articles, we divided the search into awareness, solutions, and both. Awareness systems are used to make the user conscious of a certain problem or situation, while solutions is for the presentation of a system that solves a certain problem. Some systems can have both awareness and solutions. We classified 29 articles as awareness, 26 articles as solutions, and only four articles as solution and awareness. The solution and awareness articles had two different ways of presenting information. Feelbook used a notification to make the user aware of their cyberbullying posts and at the same time, it is considered as a solution since it uses interventions when posts of cyberbullying were posted or when reporting to adults about these posts [76].

For Rafigh, mushroom growth was used for awareness of the measure of speech of a child and as a solution to motivate the child to speak for longer periods [77]. Regarding empathic car interfaces, awareness was by sharing information about the time required before arrival at the destination, while the solution was presented based on the behaviour of the user by providing breathing manoeuvrers to reduce stress and increase mindfulness [62]. The same is true for when the car gives biofeedback to the user to make them aware of their state and then provides solutions to problems in certain situations [65].

##### Automation Level Changes

For the automation level changes, we attempted to investigate whether any of the systems was able to automate itself based on a certain situation. This feature is still not included in the empathic systems presented. Only two articles mentioned a self-driving mode for their future work [62,65] by activating it to help calm the driver, yet the automation was not mentioned: we do not know if the car takes the decision to activate the self-driving mode or if it tells the user to do so.

##### Design and/or Implementation

For the design and/or implementation, we attempted to determine whether the articles were already proposing a solution that had been developed with a certain prototype, or whether it was still in the design phase and was the creation of mockups with the design elements. We found that 29 articles had already existing prototypes, thus, belonging to the implementation cluster, while 18 articles proposed a design for their solution, and 12 presented the design process of their solutions and their implementation. We attempted to group the studies based on the output shape and found similar groups between the three clusters.

The articles with implementations are grouped into applications [40,42,46,55,71,72,76,78,79,80], digital companions [45,53,58,70,81,82], robots [59,61], living or digital interfaces [62,77], visualisations [43,44,73,83,84,85], physical products [39,57], and frameworks [86]. For the design and implementation, many groups are similar to the implementation only, as applications [41,49,87], agents [48,54,68,88], products [63,74,89,90], and visualisations [50,89]. For the design, we grouped the articles into behaviour design [75,91,92,93,94], machine and application design [47,52,56,60,65,69,95,96,97], visualisations [67], and strategies and approaches [51,64,66].

##### Field

The annotation in the diagram is ArticleID_field, where the number is the article ID and field is the sector in which the article tackles the problem.

**Figure 9:**
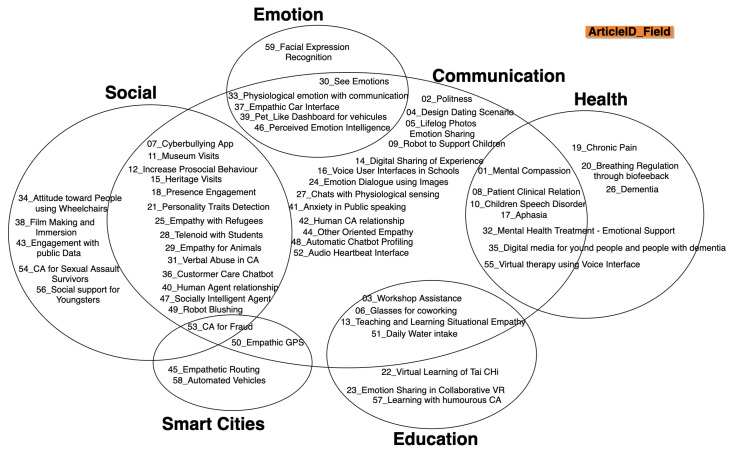
Distribution of articles over the fields of usage.

We classified the articles into six important fields: Communication, Social, Health, Emotion, Education, and Smart Cities with some intersection between them. The fields are represented in Figure 9. The main field is Communication with intersections with all other fields, with 45 articles out of 59 articles, while 13 articles were designed and/or implemented for Communication only. Social and Health were well-developed with 20 articles within the Social sector and 10 for the Health sector. This leaves the Emotion sector with six articles of which five were intersected with the Communication field, seven with the Education field, and four with Smart Cities. This diagram shows that the latest studies were not only focused on the communication between machines and humans but also started to intersect with other domains showing defined scenarios. This shows that the machines today are entering a new way of performing; the machine should be able to adapt not only to the user but also to the environment and the scenario in which it is present.

##### Assessment

The assessment of any feature is crucial because it shows the way the study was evaluated and reveals the results through statistical analysis. Since empathy is still a complex concept, we attempted to extract how people evaluate their systems. We search for quantitative and qualitative calculations of empathy as well as other types of assessments if empathy itself was not tackled.

- Quantitative Calculations of Empathy
  Quantitative calculations of empathy are important to be able to quantify its level; however, the research on quantifying empathy is still immature. Thus, finding studies that calculate the empathic level is almost impossible. However, relatable analyses can be found in articles, such as the calculation of empathic concern [43] or a personalised questionnaire assessing the effects of empathy [40] both with a seven-point scale. The Self Assessment Manikin (SAM) questionnaire was used with the Markov Chain Monte Carlo (MCMC) once [84]. Another example is the compassion scale [98], which was used as a trait of empathy [83]. “PANAS”, the Positive and Negative Affect Schedule, was used to obtain personalised information from the testers [91]. An important method that was used is the empathic accuracy task [44] where the testers attempted to predict the feeling of the main participant in a particular situation.
  Personalised questionnaires were used as well as a quantitative measure where the participants self-reported their level of empathy with characters before and after the experiment [49,89], where, for the chatbots, the level of empathy was calculated based on the frequency of empathic words used [64].
- Qualitative Calculations of Empathy
  For the qualitative calculation of empathy, we extracted the methods that were used to evaluate empathy; however, since the research is still underdeveloped, we attempted to gather close and important factors. These methods can be summarised as human annotators [43,88], questionnaires [40,45,49,89], free inputs [43], and the most-used method, interviews [41,44,51,55,83]. Humans annotated the empathic level of response and passionate level [88], as well as, on the other hand, their empathic concern and personal distress [43] based on the adjectives of Batson’s list of emotions [99]. A self compassion questionnaire was used in another study [45].

##### Other Types of Assessment

For other types of assessment, we attempted to extract the most important ways that the systems were evaluated for multiple purposes. Multiple studies used predefined questionnaires, such as NASA-TLX [75,84], MeCue questionnaire [58], self assessment measures [71], presence questionnaire/narrative engagement scale [83], inclusion of others in self-scale (IOS) [91], Affective Benefits and Costs of Communication Technology (ABCCT) [41], depression severity (PHQ-9)/anxiety severity (GAD-7) [96], Perceived Emotional Intelligence (PEI) [68], the likeability and perceived intelligence subscales [87] or the four GodSpeed indices [54], Intrinsic Motivation Inventory (IMI) [87], the Academic Motivation Scale (AMS) [87], the Humour Styles Questionnaire (HSQ) [87], and Film Immersive Experience Questionnaire [85]. More complicated methods were also used, such as fNIRS—functional Near-Infrared Spectroscopy [71].

Other methods included interviews with participants [41,46,49,50,70,72,74,80,85,90,92,94,95,96,97] or experts on the topic [54,92]. The most-used method was the personal questionnaire [39,41,46,48,49,54,56,57,58,66,69,73,89,91], which included questions about feelings [44], game experiences [95], response quality [88], the perception of responsibility and justice [43], awareness [40], and trustworthiness and privacy concerns [48].

In addition, other types of assessments were used, such as the angle of vision of two participants [91] and the breathing traits [84], or by clustering the voice recording based on the Russell model of the positive–negative model [62].

### 4.3. Results Taken from Articles: What Did the Author Find as the Result

We include a list of all the findings of each of the articles in Table 2.

## 5. Discussion

This systematic review provided a global view of the emerging area of empathic and empathetic systems within the CHI conference scope between 2011 and 2021. We now discuss the findings based on each of the features presented. We attempted to draw conclusions on what empathic and empathetic systems should be like, what it should detect, and what type of response it should generate.

As shown in the state of the art, there is still no standardised definition of empathy for researchers to follow. Due to this diversification, many questions emerge on the unification of the definition of empathy, the importance of understanding the researchers point of view, and the way it is implemented. One interesting aspect confirmed from this analysis is that empathy needs two interacting parties and that it is about feeling and understanding the other. We present two definitions to summarise the distinct findings:

Short Definition:Empathy combines factors initiated by an interactive sequence of detection and responses between two parties.

Long Definition: Empathy is a combination of interactive sequences that consists of being able to detect and understand the other parties’ feelings, understand the context and the situation, and analyse and respond to those feelings accordingly on three axes: affective, cognitive, and compassionate. The three axes of empathy are each defined by detection and response factors:

(1) Affective, where a person instantly evokes the same feelings and emotions of another because of past experiences, the detection is based on empathic matching of emotions, while the response is based on empathic concern.

(2) Cognitive, where a person can mirror and understand the feelings of another without having past experiences where they actually felt the same but rather being able to connect multiple experiences. This is more based on the instant analysis of the context and situation by the receiver and their projection into the perspective of the sender. The detection is based on imagining the self in the position of the receiver, and the response is based on imagining the other perceptions to our response.

(3) Compassionate, where a person has an understanding of the sender context and situation without actually feeling them or connecting to it. The detection is based on assisting the other by connecting and focusing and regulating self to create a safe distance with the other. The response is based on helping others to find a solution and regulate their emotions.

In the following, we present certain statements concluded from this systematic review on empathic systems, discuss the drawbacks, and present gaps that are yet to be filled.

An empathic system should be designed and implemented with detection and response sequences.

From the research on detection and response modalities, we notice that not all systems consider the cycle of detection and response. Multiple articles worked on one defined modality of response [40,42,43,52,53,55,56,72,75,76,77,80,82,87,95] or on one modality of detection [41,51,66,78,79,86], while others built a multi-modal interactive system. The consideration of the detection/response sequence was tackled by 42%—25 out of 59 articles—see Figure 4. This percentage was unexpected; our preliminary prediction was that we would have a lower percentage of studies including this factor. Multiple studies [39,47,48,54,57,59,60,61,62,63,64,65,67,68,69,70,71,73,74,81,84,88,91,94,97] designed and built their system to detect human features and to be able to respond through one or different types of modalities. We notice that most of these systems belong to the implementation [39,57,59,61,62,70,71,73,81,84] cluster—10 out of 25 articles—while two belong to the design and implementation [48,54,63,68,74,88] cluster—6 out of 25 articles—and nine studies belong to the design [47,60,64,65,67,69,91,94,97] cluster—9 out of 15 articles.

An empathic system should be designed and implemented with a multi-modal verbal and non verbal detection and response modalities

An empathic system should be able to detect, analyse, and understand every detail of a human in addition to being able to communicate the best response through a response modality that fits best. Therefore, verbal and nonverbal communication are important factors to take into consideration when building empathic systems. For the detection, most articles used nonverbal detection (21 articles) and 17 articles used verbal detection—see Figure 5. For nonverbal detection, the most-used modality was physiological signals [41,44,47,51,57,65,69,71,79,84,86,91], such as Electrodermal Activity (EDA) [44,51,69], Heart Rate (HR) [86], and galvanic skin response [57]—12 out of 32 articles. The second-most used modality was facial expressions [39,47,54,59,61,63,67,73,74,78]—10 out of 32 articles. Third-most was text [47,64,66,70,73,81,88,94,97] and voice [47,48,54,59,60,61,68,94,97] with 9 out of 32 articles. Fourth was voice to text [44,47,48,54,59,62,68]—7 out of 32 articles. Finally, body language [47,67] was the least used in the project with only two articles—2 out of 32. For the response, verbal responses occurred in 38 articles out of 52 while nonverbal responses occurred 27 times. The most-used modality was visuals [39,43,45,47,49,50,52,53,54,55,56,57,62,63,65,67,71,74,77,83,89,90,91,92,93,95]—26 out of 52 articles. The second-most used was voice [39,42,46,47,48,49,50,54,59,60,61,62,68,69,72,74,82,83,89,91,94,96,97]—23 out of 52 articles. Third-most was text [40,45,46,47,58,64,65,70,73,75,76,80,81,83,87,88,89,92,94,96,97]—21 out of 52 articles. Fourth-most was text-to-speech, which is another important modality for response but was used only seven times [46,47,54,58,59,68,71]—7 out of 52 articles. Finally, music [47,65,74,84,85,90,93]—7 out of 52 articles—and light [47,65,67,84]—4 out of 52 articles—were the least used. Interesting and unique features were used when developing response modalities. For example, in the voice response, heart beat sounds were used by the receiver to increase consciousness about the transmitter’s state [91]. Another important response is images with the real-time feedback of Social Signal Processing (SSP) [67]. Growing mushrooms were used as well to help children with speech problems [77]. Others used screen games [55,95] and artistic pictures, such as drawings drawn by orangutans [52] and abstract data visualisation [57]. We see that empathic systems are starting to grow in other fields and are being developed in different ways. Different modalities are implemented depending on the field of usage. In detection, many studies are still to be made; body language is one of the least developed modalities. In response, light and music are the least developed, yet music can affect humans consciously or unconsciously, the same as light, which can be used as a discrete method to affect the user subconsciously and using peripheral methods.

An empathic system should be designed and implemented to detect the context of the scenario, adapt, and change its behaviour techniques

From the analysis, we notice that not many articles considered the context. The detection of context is important because it helps the system understand the global situation of the user. This detection can be made on different axes: on a behavioural level [48,54,58,59,60,61,62,68,70], on an emotional level [39,47,65,66,67,69], or on a visual level [63,71,72,73,74]. The context detection should not be limited—it should be expanded to other types of context as a situational context, for example. Understanding the situation can help the machine to adapt itself. Adaptation is an important feature that needs to be included in any system. This adaptability should help the machine communicate the information to the user according to the context of the situation. With adaptability comes the change of behaviour techniques, where the machine should be able to switch between modalities. When the human, for example, is not able to talk to the machine, the latter should adapt its behaviour by sending the output through a different modality, such as by text instead of voice. The change in behaviour should not be intrusive. We noticed that sometimes interruptions can have a negative impact on the user when they exceed a certain level [75]. The same is true for movements. In the case where a machine is employing movements, it should not bother the user from the task they are doing. The machine should understand and set limits for its behaviour.

An empathic system should be designed and implemented as for a goal of awareness and solution in every domain.

As is clear in this review, the fields of empathic systems are still concentrated in six important sectors as seen in Figure 9: communication, social, emotion, health, smart cities, and education. These fields need to be expanded to reach other types of sectors, including industrial, media, telecommunications, journalism, public services, etc. Empathic system development, when designed or implemented, needs to consider its ability to provide awareness or present solutions to the user. This can be through informative methods based on certain rules defined to notify users about a certain danger or through functions to help the user find their way to surpass certain problems.

An empathic system needs to be evaluated based on its empathic level through qualitative and quantitative assessment to be able to improve the empathic interaction quality

A significant evaluation of the system can improve its quality and accuracy. For that, the use of qualitative and quantitative assessment is a must. Currently, most studies use a personal questionnaire due to the lack of standardised methods for empathy evaluation. For the quantitative evaluation, we notice that an empathic concern calculation [43] exists, yet it was only used by one study. We did not find a quantitative empathy calculation that was widely used. As for the qualitative calculation, multiple types of questionnaire were used, in addition to human annotations; however, as with the quantitative measure, we did not find a standardised questionnaire. From here, we raise the problem of standard empathy assessment, whether quantitative or qualitative, and the lack of research on these types of assessments. It might be that empathy is still vague and undefined.

### Empathic Interface Interaction Modelling—Real Life Examples

This systematic review provides an overview of the emerging research and development of empathic systems. After the identification of the important parts and major modalities of interaction in an empathic system, we now present examples of real life scenarios where empathic interactions can be applied. We imagine that the empathic user interface will be the next evolution of interfaces where emotions are included.

The role of an empathic user interface—EUIs is to be empathic with the user by sharing their positive feelings with them and by helping them out of their negative ones. For this to occur, the machine needs to have the crucial traits highlighted in this systematic review. The machine needs its detection–response sequence, it needs to adapt, and finally it needs to change its behaviour based on the situation.

**Figure 10:**
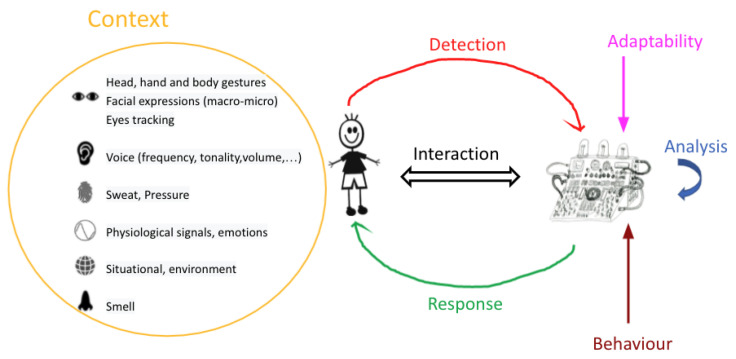
Empathic user interface.

Therefore, an empathic interface should be able to detect and predict the user’s emotions by analysing voice tonality and frequency, facial expressions, physiological signals, or even the vocabularies used in a text conversation with the interface. After the analysis, the system should empathically and appropriately respond to the user via text, voice, music, visuals, or even lights. The general concept is represented in Figure 10. Moreover, the interface should possess the ability to adapt to external circumstances, such as when interacting with several people instead of one person as seen with Murphy the robot when discussing with a group of children [59].

Furthermore, since this interface is of empathic nature, it should also be able to change how it interacts and how it responds to the user based on the situation: if the emotional state of the user requires relaxing music in order for them to feel better, the system should shift from replying with a text to playing or suggesting a song, for example. On another note, the system should be environmentally aware, therefore, it should be able to alert the user when needed by interrupting them [75] and attracting their attention using sound [62], lights [84], or texts or movements [74].

As already mentioned, EUIs are an important and needed type of user interface. Even though a fully developed model for these interfaces has not been released, many concepts and applications for the EUI are emerging. In the following, we will propose ideas for real world applications that can be adapted and set to be empathic.

ChatBot:Chatbots are the most famous EUI applications since people tend to feel less intimidated when sharing their thoughts and feelings with a robot instead of a human. A chatbot detects the context by analysing the words typed by the user during an interaction, and then it can interfere and fix typos and grammatical errors, set reminders for events, etc.

**Figure 11:**
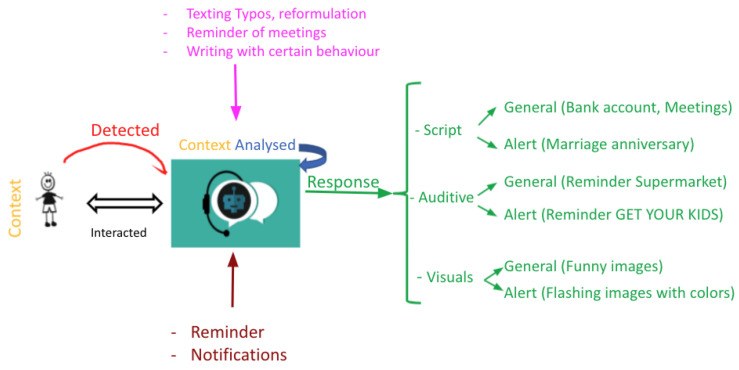
Example 1: Chatbot.

The chatbot also predicts the emotional state of the user based on the conversations, and it can respond with text, audio messages, or media files according to the situation at hand. Additionally, in the case of an urgent matter, the system should adapt its response and alert the user by sending them notifications, vibrating, or even minor electric shocks in order for them to halt their actions and focus on what is important (Figure 11).

**Figure 12:**
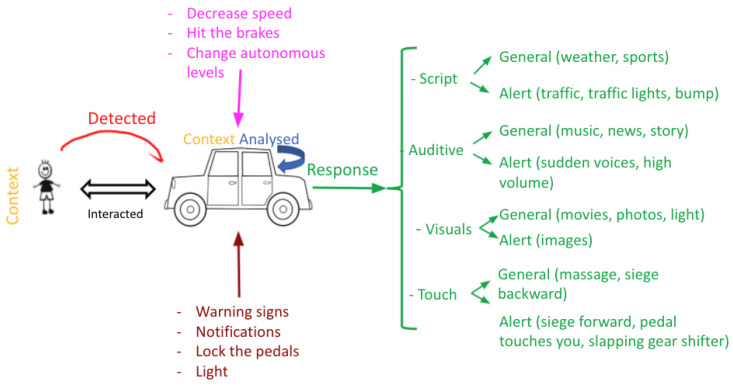
Example 2: Autonomous car.

Autonomous car: An autonomous car is a popular EUI example, mainly due to the importance of ensuring safe driving and lowering the risk of car accidents. The car detects the driver’s facial expressions, voice tonality, physiological signals, and even body perspiration, and after that, the system analyses these inputs to respond according to the analysis. The responses can be scripted, auditive, visual, and tactile. Furthermore, the autonomous car should be able to adapt itself and alert the driver in the case of an emergency or unusual circumstances. By sending warnings in the form of text, images, sounds, and lights, or by taking initiatives, such as decreasing the speed, hitting the breaks, or locking the pedal, the machine should adapt depending on the emergency level (Figure 12).

Spotify: Spotify is a popular worldwide audio streaming service, and transforming this into an empathic interface can be very useful, since many people turn to music when feeling sad, happy, anxious, stressed, etc. Therefore, imagine how beneficial it would be if their own streaming application could suggest music or play songs based on their moods and emotions.

**Figure 13:**
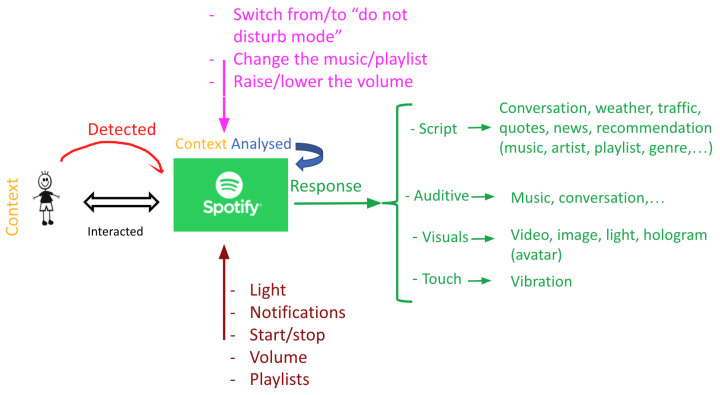
Example 3: Spotify.

Spotify will detect the listener’s feelings from the music they listen to, their facial expressions, and voice, and after analysing the context, the app can respond to the user’s emotions by recommending music, playing videos or images related to the mood or to help lift it, or by starting a random conversation to distract him. Additionally, Spotify should adapt by sending notifications, changing the volume, music, and vibrating as a way of grabbing the user’s attention if necessary. For example, if their physiological signals show high peaks of stress levels, Spotify can vibrate to notify the user of the danger, which is an awareness function, and then propose a certain song that has been tested to lower the user’s stress as a solution function (Figure 13).

## 6. Conclusions

Lately, particularly with the fast development of artificial intelligence and machine-learning models, research has shown an increased interest in designing and implementing a machine that is able to understand humans and respond to them. With the advancement of affective computing, emotion detection is becoming easier and receiving interest from the research world. Yet, a great deal of work is still missing to connect detection and response and to build empathic models. This review collected the latest articles that mention empathic and empathetic keywords from the CHI conference between 2011 and 2021.

We attempted to define the most important points that researchers have been working on lately. We extracted the most-used features to create empathic interactions from the machine-detection process until the response operation. The modalities included in this research determine the factors that will be used in the future to create empathic machines. Furthermore, we attempted to determine if the systems created had a futuristic vision for context detection, adaptability of the system, and behaviour changing.

We focused our research on the fields in which these systems are used to understand their purpose for the future, from awareness or solution vision and whether the system was already implemented or if it is still in the design phase. Moreover, since empathy is still an ambiguous topic in research, we gathered the different definitions used and proposed a common definition. In addition, empathy assessment was collected to investigate the best practices.

Finally, we included real-life examples to raise awareness regarding the significance of empathic interfaces in daily life to, thus, be taken into consideration in future studies. We noticed that there are multiple gaps that still need to be filled in the next few years to have a complete, empathic machine. This study will be used as a foundation for the development of affective and empathic interfaces in the near future.

## Figures and Tables

**Table 1:** Occurrence of “empathic” and “empathetic” in the chosen 59 articles per year.

Year	Empathic	Empathetic
2011	1	6
2012	0	1
2013	12	0
2014	3	4
2015	0	1
2016	11	4
2017	32	28
2018	1	93
2019	30	18
2020	137	5
2021	87	59

**Table 2:** Article Detail Data Sheets of 59 papers including the ID, year, design or implementation, and the findings of each of the systems.

ID	Year	Ref.	Design or Implementation	Findings of Each Article
1	2019	[45]	implementation	Chatbots help increase humans self-compassion by caring for it.
2	2013	[75]	design	Reduction of interruptiveness depends on the cognitive workload and on the type of interruption (polite vs. neutral). Higher mental concentrations are needed for polite messages.
3	2019	[58]	implementation	Providing positive experiences at work with companions.
4	2016	[47]	design	Design world where empathy is the main component of communication.
5	2018	[71]	implementation	Activation of empathic and emotional behaviour of the receiver can be made through sharing lifelog photos of the transmitter.
6	2016	[39]	implementation	Development of Empathy Glasses. Efficiency of collaborations for task execution.
7	2016	[76]	implementation	Development of an application to stop cyberbullying and promote awareness and positive behaviour.
8	2013	[67]	design	Usage of visual feedback of nonverbal cues to increase clinicals-patients communication.
9	2016	[59]	implementation	Development of a social robot to help kids in doctors waiting room.
10	2014	[77]	implementation	Development of a mushroom prototype to help kids with speech disorders.
11	2019	[46]	implementation	Development of an application for changing the gift action by replacing it with a photo and an audio to enhance relation between transmitter and receiver.
12	2017	[43]	implementation	No empathy level or prosocial behaviour difference between anthropomorphized and standard graphic.
13	2014	[51]	design	Designing technology for situational empathy for counselling students.
14	2019	[44]	implementation	Digital sharing of text or physiological signals on media impact the receiver perception. Narrative text has a positive impact on the valence of the receiver on the transmitter experience by increasing empathic accuracy, while adding biosensory information has an opposite negative effect.
15	2016	[72]	implementation	Creation of an interactive multi-narrative soundscape in museums for historical contents that impacts the visitors’ experience in a personal way.
16	2019	[60]	design	Presenting a design space for using Voice User Interfaces (VUIs) in inclusive education. Suggesting a set of scenarios that show the uses of VUIs in inclusive education. Proposing an example of a prototype application that materialises one of the proposed scenarios.
17	2011	[40]	implementation	Development of a novel system and model that enables users to experience the communication-distorting effects of aphasia which increases awareness, empathy and understanding toward patients with aphasia.
18	2018	[83]	implementation	High immersion viewing platforms do not increase empathy and narrative engagement compared to low immersion platforms.
19	2016	[95]	design	Development of a game design that enhance understanding and empathy for chronic pain patients.
20	2018	[84]	implementation	Development of a wearable device that collects breathing patterns and provides feedback modalities, such as audio, visual and haptic. Participants’ imitation of the breathing pattern provided, helps them understand the emotion behind it.
21	2019	[79]	implementation	Proposal of a framework that objectively and accurately detects a personality trait using physiological responses to external stimuli.
22	2016	[53]	implementation	Eysenck’s theoretical model is useful for generating Animated Pedagogical Agents (APAs) with different personality types. The learning experience and performance are highly influenced by the personality and the emotional feedback of the APAs which can be expressed using upper-body movements.
23	2017	[91]	design	Gaming experiences strongly influence heart-rate cue. Insignificance of heart-rate effect and a low statistical power due to the current experimental setup.
24	2018	[73]	implementation	Development of a image-grounded conversational agent that generates more emotional, informative and relevant dialogues due to the use of visual sentiments, facial expressions and scene features.
25	2017	[55]	implementation	Development of a mobile app to enhance empathy toward Syrian refugees.
26	2019	[92]	design	Designing fiction probes helps gain insights from users and produces data on complex and sensitive topics.
27	2017	[41]	design and implementation	Development of a mobile chat application that creates empathy between interlocutors by providing a context cue and encouraging engagement in chat activity.
28	2012	[61]	implementation	Development of a tele-operated humanoid robot that positively affects group work between schoolchildren due to its limited functionalities, by awakening their inner caregivers.
29	2017	[52]	design	Empathy, in all its forms, is evoked in human observers of animal-computers interactions.
30	2018	[66]	design	Development of an alternative evaluation method for psychologists to conduct larger-scale emotion studies.
31	2019	[81]	implementation	The agent’s response style to verbal abuse significantly affected the user’s emotions by reducing aggression. The agent with an empathic response rendered the participants less angry and more guilty in comparison to the other responses (avoidance and counterattacking).
32	2018	[96]	design	Guided and unguided chats are useful for reducing anxiety; however, guided chats provoked undesired attention on troubles in some cases, while unguided chats diverted the attention from the trouble instead of providing emotional support.
33	2015	[57]	implementation	Development of a wearable device that uses galvanic skin responses and heart rate to show the user’s emotional state.
34	2014	[56]	design	Embodied persuasive games helps with removing barriers between able-bodied people and people with mobility impairment.
35	2018	[80]	implementation	Design of a mobile application that improves relationships and encourages conversations between young people and their older relatives suffering from dementia.
36	2018	[88]	design and implementation	Development of a tone-aware chatbot able to generate responses considered more empathetic than those by human agents.
37	2019	[62]	implementation	Identifying the origin and frequency of emotional triggers that affect humans while driving. Suggesting methods to overcome negative triggers and stabilize the driver’s emotional state.
38	2017	[85]	implementation	The viewing platform and the use of headphones significantly influenced the immersive experience, while the platform type did not have an impact on all aspects of the immersion.
39	2016	[74]	design and implementation	Creation of a pet-like dashboard system considered as a driving companion which helps drivers communicate positively.
40	2021	[63]	design and implementation	Creation of an eye looking camera prototype able to follow and imitate human eyes.
41	2020	[82]	implementation	Development of a prototype to help with reducing anxiety in public speaking using Amazon Alexa by implementing a coach for cognitive intervention.
42	2020	[42]	implementation	Development of a conversational agent prototype that uses empathic nonverbal vocal cues to prevent negative feelings and establish a relationship with the agent by increasing intimacy similarity
43	2020	[89]	design and implementation	Creation of personalised film to increase empathy with nonexperts users by transmitting the data in an easy comprehensible way.
44	2020	[50]	design and implementation	Creation of virtual reality games to improve other-oriented empathic behaviour.
45	2021	[86]	implementation	Creation of a framework that uses Heart Rate signals to guide drivers on a personalised route.
46	2021	[68]	design and implementation	Creation of a conversational agent that is acoustically aware of the user’s voice, making it more emotionally intelligent than other agents
47	2021	[54]	design and implementation	Creation of an avatar with features as facial expressions and lip syncing. It can understand the conversation and the expression style of the user and is better animated than agents that do not have.
48	2021	[64]	design	Design of a framework to evaluate chatbots which helps chatbot creators test multiple features and improve iteratively their chatbots.
49	2021	[93]	design	Design of an ATM robot that can blush or express embarrassment through noise and movement through the opening of the machine.
50	2020	[69]	design	Design of a GPS voice system that can adapt its voice to the user arousal, valence and stress levels.
51	2021	[90]	design and implementation	Creation of an interface to remind users to drink water by sharing emotional responses. Impacting positive behaviour and health.
52	2021	[49]	design and implementation	Testing the heartbeat sound as an empathic response. Showing that heartbeat sounds can increase the ability to feel what the other is feeling.
53	2021	[48]	design and implementation	Creation of a conversation agent that can discuss with the user to stop fraud and risky transactions by asking for more transaction details.
54	2021	[94]	design	Modelling a conversation agent for sexual assault survivors that can provide informational and emotional support
55	2021	[97]	design	Conceptualisation of a voice user interface to integrate it in virtual therapy and identification of its advantages and limitations.
56	2021	[70]	implementation	Development of a chatbot that can provide informational and emotional support to young people.
57	2021	[87]	design and implementation	Development of a conversational agent with humour abilities to improve learning experiences and relationship with users
58	2021	[65]	design	Designing empathic interactions in cars through the usage of multiple detection and response modalities
59	2016	[78]	implementation	Development of a real time facial expression recognition system for multiple people with minimal false detection.

## Data Availability

The data presented in this study are available on request from the corresponding author.
